# Conservative Approach and Management of Complicated Lung Abscess

**DOI:** 10.7759/cureus.31910

**Published:** 2022-11-26

**Authors:** Benoit Boucher, Doina Virlan, Venkata Buddharaju, Sanket Chaudhari

**Affiliations:** 1 Pulmonary and Critical Care Medicine, Weiss Memorial Hospital, Chicago, USA; 2 Internal Medicine, Saint James School of Medicine, Chicago, USA

**Keywords:** abscess, drainage, infection, lung, pulmonary

## Abstract

A 72-year-old male presented to the ER with three days of productive cough, shortness of breath, and generalized weakness. Chest X-ray showed right lung opacity in the lower lobe. Chest CT scan showed consolidation in the superior segment of the right lower cavity with air-fluid level extending to the pleural and chest wall, suggestive of lung abscess with loculated empyema and thickened pleura. The patient received antibiotics and CT-guided aspiration of blood-tinged fluid followed by two weeks of drainage via a transthoracic catheter. There was a near-complete resolution of the opacity and closure of the lung abscess on follow-up chest imaging. The patient clinically improved with resolution of the cough and dyspnea. Workup was negative for bacteria and acid-fast bacilli (AFB). The purpose of this paper is to review short-term and long-term management, approach, and consideration to be taken while facing a pan-negative etiological workup of a complicated abscess.

## Introduction

Lung abscesses are defined as a circumscribed area of pus or necrosis in the pulmonary parenchyma caused by microbial infection. They are classified as primary (from direct infection of the pulmonary parenchyma in healthy patients) or secondary due to a predisposing condition such as hematogenous spread through septic embolization (right-sided endocarditis), bronchial obstruction (foreign body, neoplasm), infection of a lung cyst or immunocompromised status [1]. They can also be classified as polymicrobial or monomicrobial [2]. Most often, lung abscesses arise as a complication of aspiration, especially amongst alcoholics which then progresses to tissue necrosis after seven to 14 days. Three factors play a role in aspiration abscesses: altered clearance, large inoculum size, and impaired defenses. The most common florae are microaerophilic streptococci (*Streptococcus anginosus*, *Streptococcus milleri *group, oral streptococci such as *Streptococcus mitis*) and anaerobes (*Peptostreptococcus*, *Prevotella*, *Bacteroides *[3,4]. Another mechanism is through hematogenous spread through embolization, most often from endocarditis or thrombophlebitis (catheter-induced and Lemierre syndrome) [5]. Other possibilities are superinfection of pulmonary infarcts, congenital malformations, lung contusion, or bronchiectasis that get infected. Abscesses can arise on a post obstructive pneumonia due to obstruction or from other obstructive etiology such as a tumor and aneurysm of mediastinal vessels [6]. 

Pyogenic bacterias implicated in lung abscesses are *Staphylococcus aureus*,* Klebsiella pneumoniae*, *Pseudomonas aeruginosa*, *Burkholderia pseudomallei*, *Haemophilus influenzae* type b, *Legionella*, *Nocardia*, and *Actinomyces*. Three groups of pathogens have been described which include mycobacteria such as *Mycobacterium tuberculosis*, nontuberculous mycobacteria (*Mycobacterium avium*, *Mycobacterium kansasii*, *Mycobacterium abscessus*), and parasites (*Entamoeba histolytica*, *Paragonimus westermani,* *Echinococcus*). In immunocompromised patients, the most common causes of lung abscess are *P. aeruginosa* and other aerobic gram-negative bacilli, *Nocardia*, and fungi (*Aspergillus* and *Cryptococcus*) [2,7]. Lung abscess presentation can be indolent or symptomatic but most often, patients present with fever and chills (80% of cases), productive cough (55% to 90% of cases), chest pain (20% to 35% of cases), and hemoptysis (10 % of cases) [8]. Diagnosis is usually established with imaging and supported by the above-mentioned symptoms. Two sets of blood, sputum, and catheter cultures when present may support the diagnosis but are not deemed necessary for diagnosis [9]. Chest X-ray is usually used as the first mainstay of imaging. Abscesses can then further be better visualized and characterized via CT scan. Such lesions will show as an area of cavitation surrounded by consolidation. In the acute process, the region with necrosis should be irregular in shape with a thick wall. As it progresses to the chronic process, the walls become thin and uniform as the abscess resolves [4]. Differential diagnosis includes excavating tuberculosis and mycosis [1]. Excavating bronchial carcinomas such as squamocellular or microcellular carcinoma usually present with thicker and irregular walls [10]. The appropriate diagnosis then relies on the localization of the lesion and clinical signs. Localized pleural empyema can be distinguished by using a CT scan or ultrasound [1,11]. 

In the pre-antibiotic era, mortality from lung abscesses was approximately 75% without treatment [1]. Lung abscess mortality decreased to 20% to 35% with the addition of percutaneous drainage and less than 8.7 % with antibiotic therapy alone [1,8]. Treatment initially consists of empiric antibiotics which are later adjusted based on culture results. The preferred initial empiric antibiotic therapy is beta-lactam with beta-lactamase inhibitors followed by imipenem or meropenem since it covers the most common etiologies of aspiration previously mentioned [1]. It is most important to treat the underlying bronchial obstruction if present while surgery with drainage is reserved for cases refractory to antibiotics [1].

## Case presentation

**Figure 1:**
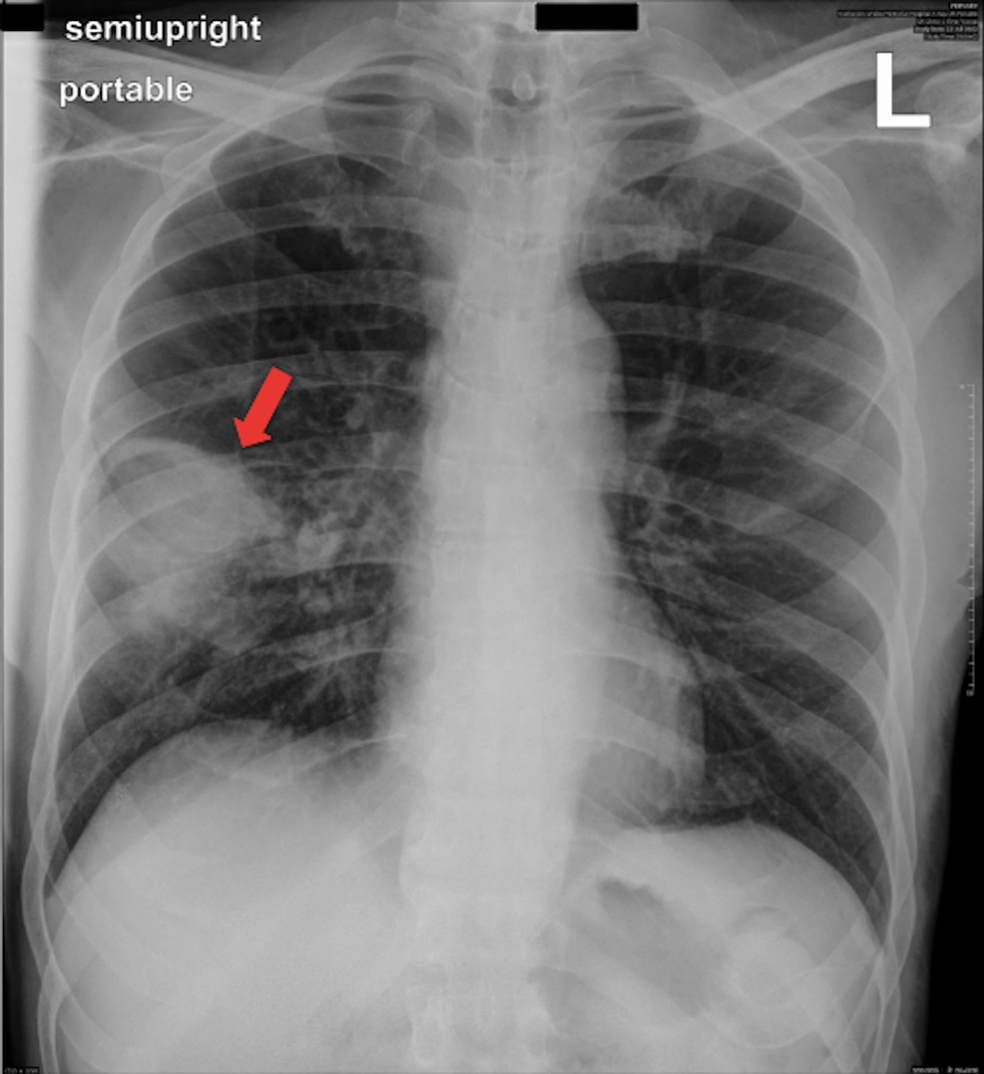
Chest X-ray on admission. The red arrow indicates the area described as consolidation.

A 72-year-old patient presented to the emergency room complaining of increasing cough with green sputum for three days as well as shortness of breath and generalized weakness. Vital signs were: heart rate (HR) 107bpm, blood pressure 151/84mmHg, oxygen saturation (SaO2) 96%, respiratory rate (RR) 20/min on room air, and temperature 37.2˚C. The patient denied chest pain, fever, nausea, vomiting, bloody sputum, dizziness, weight loss, chills, night sweats, or recent travel and sick contact exposure. The physical examination was unremarkable except for decreased bilateral breath sounds with right-sided rhonchi in the basal region, cachexia, and appearing malnourished. Oral hygiene was noted to be poor, and HIV testing and a history of tuberculosis were found to be negative. He denied any history of steroids or intravenous drug use nor ever contracting COVID-19 for which he received three doses. Relevant co-morbidities were untreated hepatitis C and diabetes mellitus diagnosed and managed during his hospital stay. His social history was notable for alcoholism to which he admitted being sober during the last four years. He evasively endorsed one to two drinks a week but didn't seem intoxicated and was alert and oriented. He endorsed smoking one pack a day for 15 years, which he quit the month prior to his visit. He admitted being sexually active with one partner. Relevant labs showed WBC 19.2K/µL, bicarbonate 17mmol/L, and lactate 1.7mmol/L. Chest X-ray showed consolidation of the right lower lobe (RLL) (Figure 1). 

**Figure 2:**
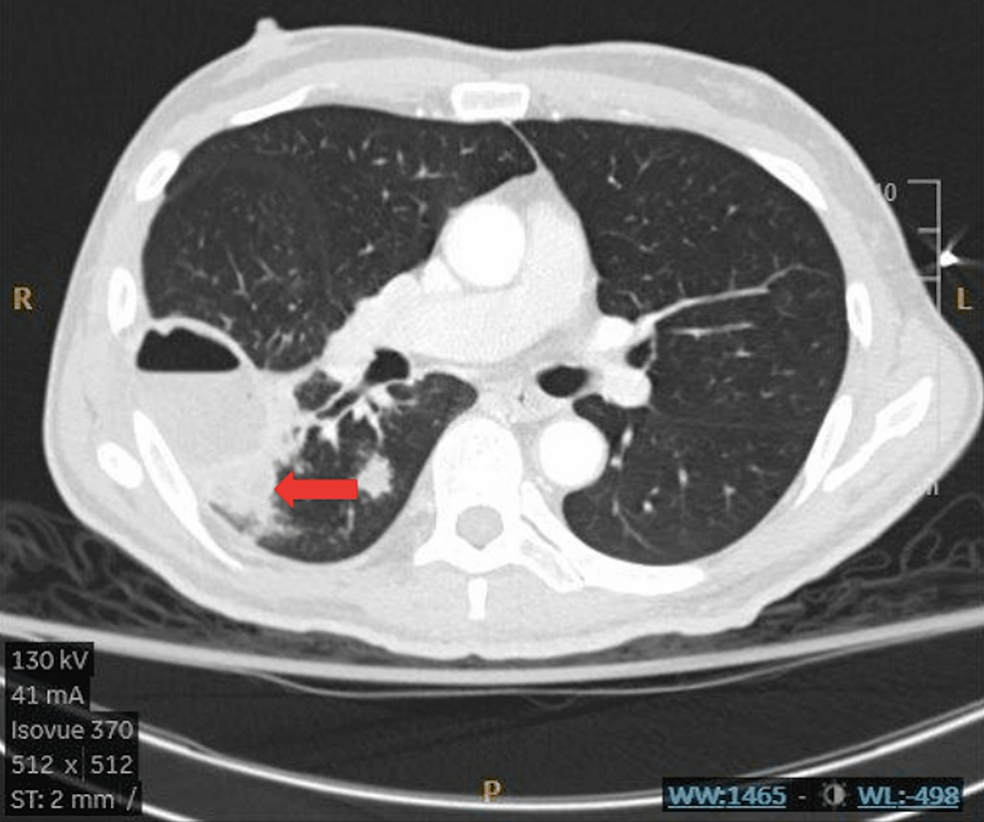
Axial chest CT showing complicated abscess. The red arrow point to the area of loculation.

**Figure 3:**
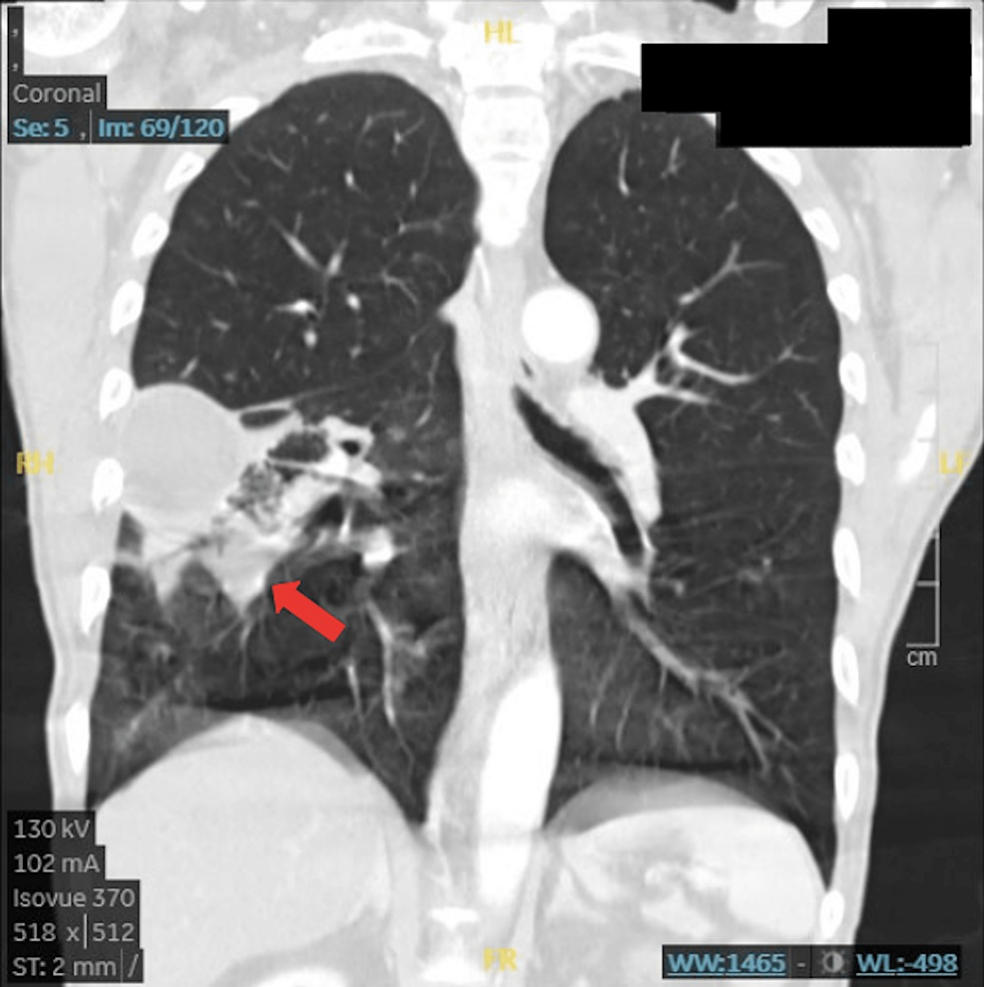
Sagittal CT scan of the complicated abscess. The red arrow points to the area of loculation.

Follow-up CT scan with IV contrast revealed a 4.5 cm x 4.1 cm x 6.5 cm cavitary lesion in the superior segment of the right lower lobe along the major fissure with air-fluid level with smooth enhancing wall and adjacent tree-in-bud opacities along with several larger nodules measuring up to 1.3 cm (Figures 2, 3). Loculated empyema was also noted.

**Figure 4:**
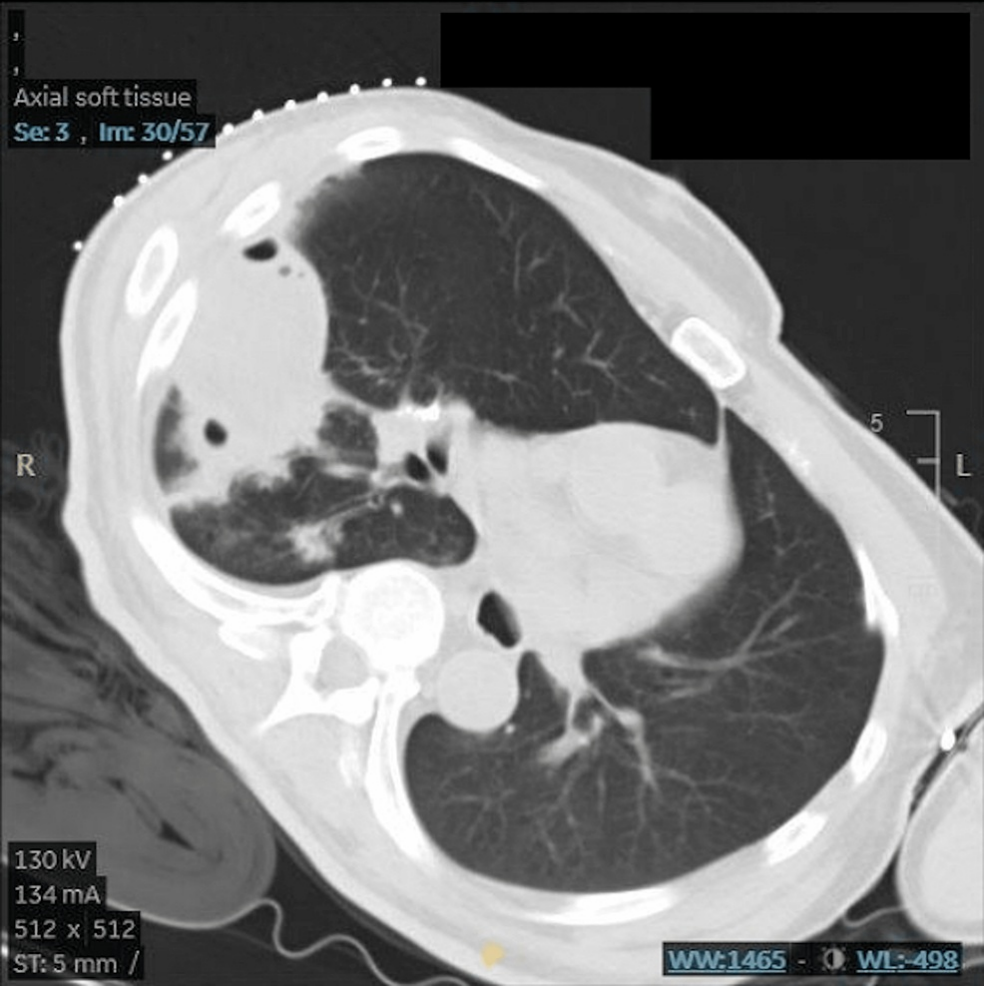
CT scan of the abscess prior to drainage

**Figure 5:**
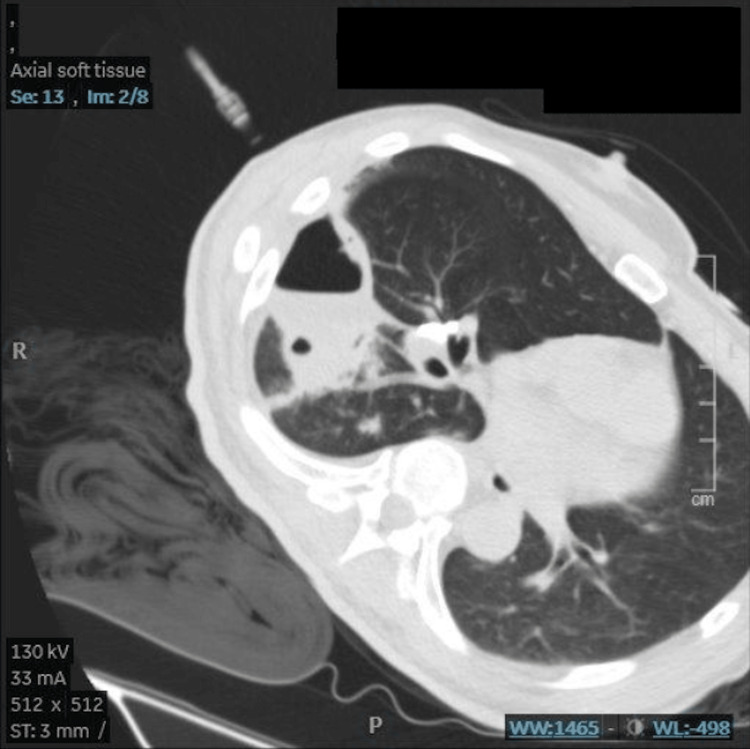
CT scan of the abscess post-drainage

Differentials at that time included cavitary pneumonia, abscess from possible aspiration, *M.Tuberculosis,* and malignancy. The patient was admitted and underwent CT-guided aspiration which drained a little less than 40ml (Figures 4, 5) of straw-colored fluid. Sputum, blood, and abscess fluid drained by CT guidance were sent for analysis and culture. The patient was treated with vancomycin 1.0g, piperacillin/tazobactam 3.375g, and oxygen support as needed. 

Bacterial, mycobacterial, fungal stains and cultures, polymerase chain reaction (PCR), Quantiferon of sputum, and abscess fluid all came back negative except for a few unspecified gram-positive cocci and *Stenotrophomonas maltophilia *in the sputum. The patient was managed with a peripheral IV antibiotic regimen consisting initially of piperacillin/tazobactam 3.375g every eight hours and levofloxacin 750mg every 24 hours for four weeks. Upon sputum culture results, piperacillin/tazobactam was changed to amoxicillin/clavulanic acid 875mg/125mg every 12 hours for two weeks. The patient also received daily IV vancomycin 2.0g to 2.5g for the first week. The patient tested negative for *Blastomyces*, positive for chronic hepatitis C virus (HCV) with an RNA count of 12,800,000 copies, and denied any treatment for this condition. The abscess fluid analysis revealed similar characteristics as exudative fluid (fluid proteins 2g/dL, serum protein 2.5g/dL, ratio 0.8, pH 6.8, glucose <2g/dL) readily pushing malignancy and infectious process on top of the differential diagnoses. Mild necrotic debris and abnormal mesothelial cells were noted on cytology and bronchoscopy was withheld due to non-communication of the abscess to the bronchial tree. 

**Figure 6:**
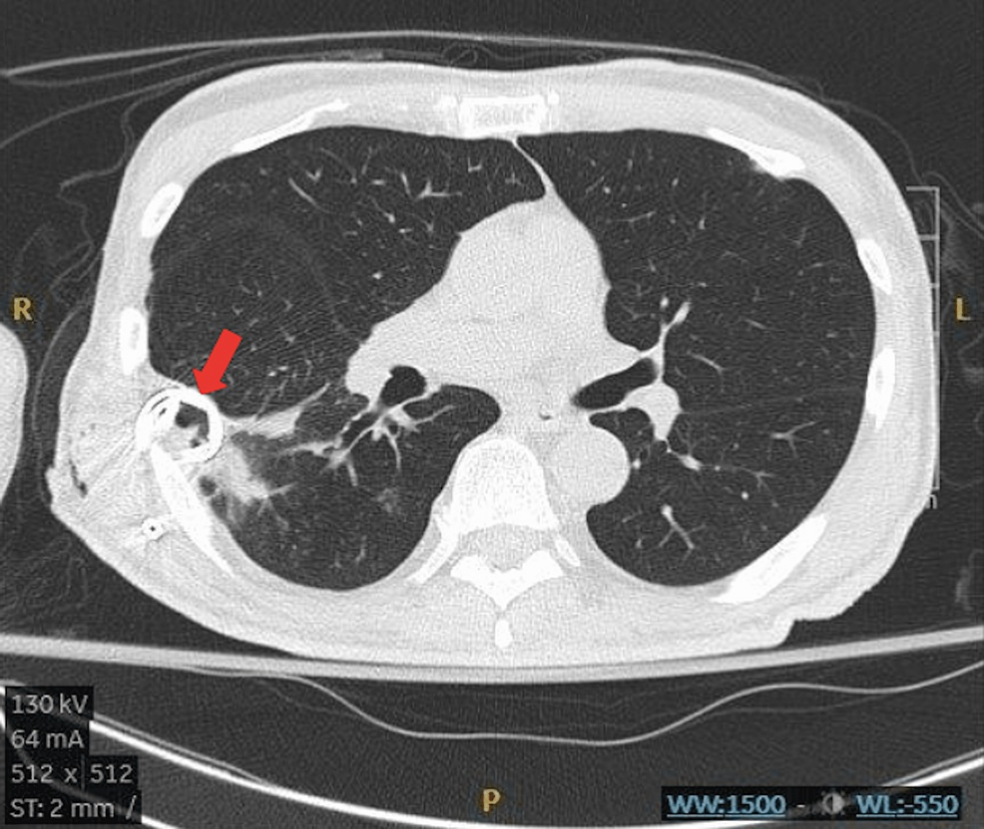
CT scan of the abscess post-catheter insertion. The red arrow points to the draining catheter.

The patient's condition partially improved over the next three days with WBC down trending (from 21.4K/µL on drainage day to 10.9K/µL), saturating above 98% on room air (previously from 96% on nasal 2l nasal cannula) without signs of end-organ failure or carbon dioxide (CO2) retention on laboratory results. Chest X-ray revealed minimal improvement displaying a 5.8cm x 4.3cm cavity with minimal consolidation. It was believed that the broncho-abscess fistula created a positive pressure system and superimposed a considerable thickness of the abscess wall making it rigid and caused it to collapse. The cardiothoracic surgery team recommended a pigtail catheter to be placed in the abscess and left there for a month. It was inserted and averaged 90ml to 100ml a day of pus and blood. A hemovac accordion drain was used to maintain negative pressure inside the drain. Follow-up imaging revealed a reduction in the lung cavity size and consolidation without residual pneumothorax or pleural effusion (Figure 6).

**Figure 7:**
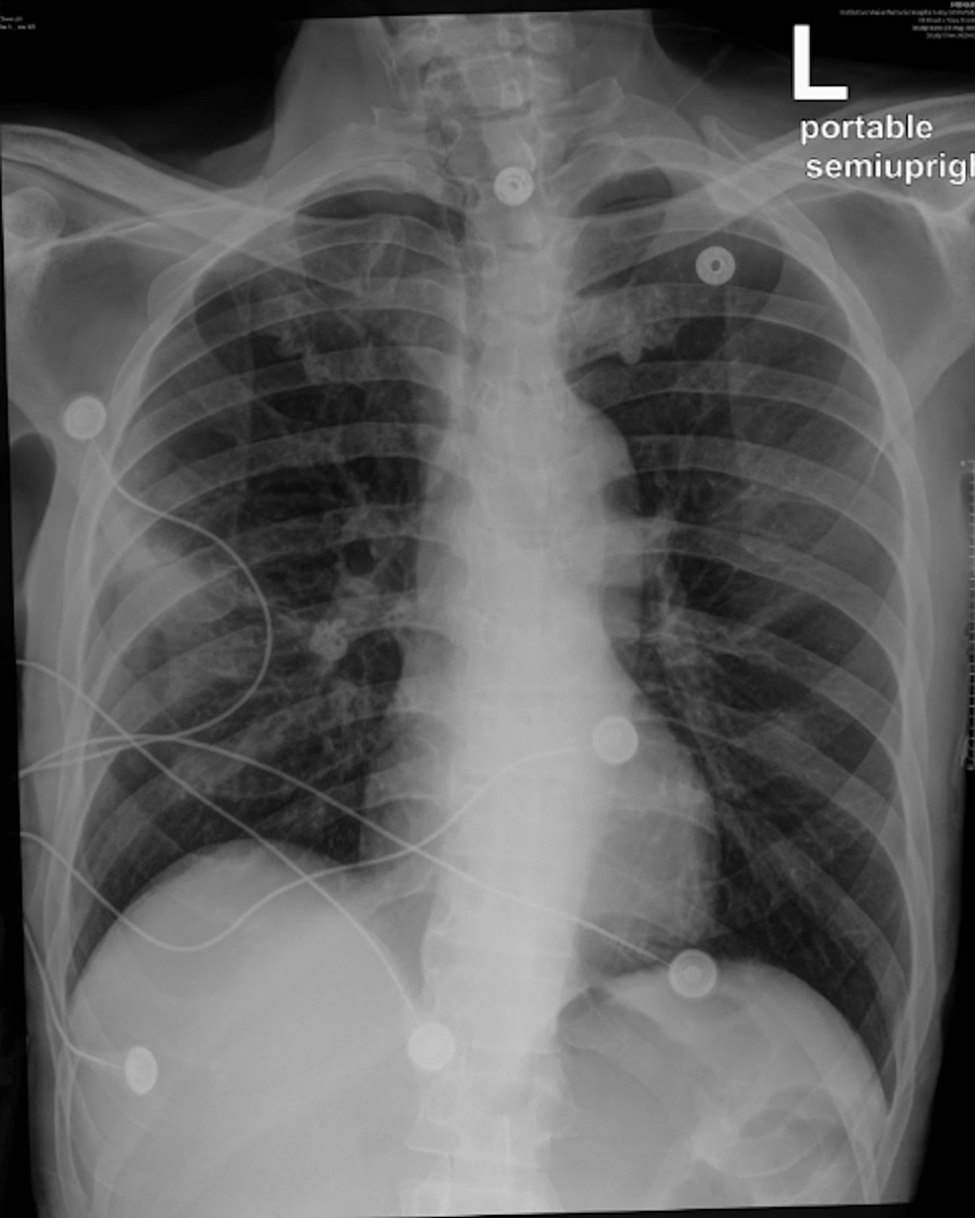
Chest X-ray post removal of the draining catheter

The patient was discharged after 35 days of hospitalization to rehabilitation. The follow-up image showed the resolution of the cavitary lesion (Figure 7).

## Discussion

Traditional management of pneumonia and lung abscesses requires initiating empiric antibiotics following the drawing of blood [1]. A review of the literature shows the rate of culture negativity of blood in the range of 49% [12]. Reasons for blood culture negativity are antibiotics given before the blood draw which tend to occur in emergent case management and aseptic abscesses. Although aseptic lung abscesses are possible such as in a pulmonary contusion secondary to trauma and pulmonary embolism [5], this patient had an incompatible story with such a diagnosis. The patient's history was significant for alcohol abuse and other comorbidities such as diabetes and a very ill cachexic appearance. This presentation with supporting laboratory values pointed towards an infectious process. Such a situation highlights the importance of good history-taking and being thorough when it comes to the chronology of events. 

We knew that the vascular instability (tachycardia) on presentation and lung abscess were interconnected but had yet to figure out if the abscess was secondary to bacteremia or the other way around. On initial presentation, none of the symptoms of sepsis were present (abdominal pain from liver failure, anuria from kidney failure, fever, shivering, confusion, rapid breathing, clammy skin, etc.) and his chief complaint was shortness of breath with green sputum coughs. A diagnosis of sepsis was conservatively established based on laboratory values and vitals (partial pressure of carbon dioxide (PCO2) 17mmHg, WBC 19.2K/µL, HR 107bpm, RR 20/min) given the patient's co-morbidities which could have lead to rapid deterioration if dissemination had occurred. These aspects subjectively implied the severity of the sepsis to be minimal in comparison to the abscess. This made us believe that the sepsis was a secondary finding to possible spillage of abscess content through fistulas that were initially too small to be observed on imaging but present at the time of admission. Secondly, the patient had poor oral hygiene and a history of alcoholism. The abscess was also found to be in the upper segment of the RLL, known to be the most common location in aspirations [1]. Liver function tests were normal although gamma-glutamyl transferase (GGT) was not performed. However, it should have been ordered to rule out recent alcohol abuse [13] which would have increased the likelihood of aspiration. The patient did drink alcohol but avoided further questions on how much and how often. No heart murmur was found related. This did not rule out the possibility of bloodborne infection, but all taken into consideration pointed toward aspiration with secondary sepsis rather than sepsis with secondary abscess of the lung. 

Most lung abscesses collapse on their own following an appropriate antibiotics regimen [1]. The interesting aspect of this case resides in the fact that it did not behave as such even with appropriately targeted antibiotics. Two possible scenarios for nonclosure arose the presence of a bronchial-abscess fistula: creating a positive air gradient to enter the cavity, and the fact that the abscess was so thick that it was too rigid to initially close. As imaging started coming back, suspicion pointed toward a possible fistula as the initial loculation appeared inferior posterior to the initial nidus with what appeared to be spillage conveniently in the same distribution. This translation of the fluids could have been explained by the patient remaining in a 45˚ Fowler’s position for an extensive period. The fistula could then have been created by the prolonged exposure to neutrophil and macrophages rich fluid which, through the actions of metalloproteinases and inflammatory mediators [14], remodeled the cavity creating the suspected fistula. Another possibility could be that the abscess was initially infected and in a manner to survive antibiotics exposure, the bacteria tried to escape the toxic environment by relocating in a new abscess. Both possibilities ultimately would have led to fistula formation. 

Usually, a complicated abscess can be managed through lobectomy [15]. Our patient was a poor candidate for such therapy given that it wasn’t a recurring problem and that any other mass being the root cause of the symptoms had previously been ruled out. The remaining option of prolonged drainage on negative pressure became interesting given the high likelihood of a positive pressure fistula causing air to enter the cavity. At the time of placement, a small right pleural effusion was found to be self-contained. We believe this effusion was uncomplicated because it was secondary to the spillage of aseptic fluid during catheter insertion. Great symptomatic improvement and cavity size reduction were achieved with the 14 French catheters on a negative-pressure accordion bag. This confirmed the possibility of a fistula pushing air into the cavity. It is believed that the mainstay of the symptoms (cachexia, weakness) was due to the abscess rather than bacteremia given the chronology of events such as conditions, symptoms resolution, initial presentation, and laboratory results. 

## Conclusions

Aspiration remains one of the main causes of lung abscesses. Clinical suspicion should be high in any patient with alcohol use or prior history of altered mental status. Although antibiotics are usually sufficient to make the abscess collapse, complications may necessitate more aggressive treatment to achieve such results. Clinicians must be thorough in their evaluation to avoid invasive surgery interventions. They must keep in mind that even complicated abscesses may be managed with minimally invasive procedures which usually improve recovery and long-term outcomes. Furthermore, clinicians must display a high level of critical thinking facing a lung abscess that is pan-negative on cultures and should question themselves if antibiotics were given before withdrawing blood for culture and analysis. Real-life emergencies may alter protocols for the greater good of the patient's health and one should review the chronology of events. Such habits may help doctors to better understand the underlying pathological process and further guide their treatments, allowing lesser delay thus reducing the risk for potential complications. Ultimately, many things can cause septic or aseptic lung abscesses and the etiology may remain unknown. Rare are those which will spontaneously spill and become refractory but in such cases, the clinician should focus on treating the abscess rather than trying to find the cause of it.
